# YouTube™ as a source of information for *Candida auris* infection: a systematic review

**DOI:** 10.1186/s12889-020-08731-4

**Published:** 2020-06-01

**Authors:** Jiangqing Huang, Shengcen Zhang, Qirong Xiao, Yingping Cao, Bin Li

**Affiliations:** 1 Department of Clinical Laboratory, Fujian Medical University Union Hospital, Fuzhou, Fujian 350001 China; 2 Fujian Institute of Hematology, Fujian Provincial Key Laboratory on Hematology, Fujian Medical University Union Hospital, Fuzhou, 350001 Fujian China

**Keywords:** YouTube™, *Candida auris*, Infection, Internet, Reliability

## Abstract

**Background:**

*Candida auris* is a novel *Candida* species, and has emerged globally as a multidrug-resistant health care-associated fungal pathogen. YouTube™ (http://www.youtube.com) as the largest free video-sharing website is increasingly used to search health information. Thus, the aim of this study was to evaluate the content, reliability and quality of YouTube™ videos regarding *Candida auris* infection, and to identify whether it is a useful resource for people.

**Methods:**

The YouTube™ was used to search systematically for videos using the keywords: “*Candida auris* infection” and “*Candida auris*”. Strict inclusion and exclusion criteria were used to select the videos. The videos were reviewed and scored by two independent reviewers and recorded the “title”, “length”, “views”, “comments”, “dislike”, “like”, “posted days” and “category of videos”. The videos were categorized as “poor”, “good” and “excellent” by the score. The DISCERN tool was used to assess the reliability of the YouTube™ videos.

**Results:**

Seventy-six videos were included in final analysis in our study. Most videos (59.2%, 55/76) had better quality. There were no statistically significant differences between groups in respect of the number of likes, dislikes, views, comments, percentage positivity, likebility, view rate and viewers’ interaction. Length and posted days were significantly associated with the classification. The videos were categorized as “educational video”, “new report”, “personal experience and blog entertainment” and “interview”. Significant differences were found in the source of videos and the characteristics of the individuals appearing in a video between the groups.

**Conclusion:**

YouTube™ has striking potential to be an effective user-friendly learning interface for people to obtain information of *Candida auris* infection.

## Background

*Candida auris* is a novel *Candida* species first reported in Japan in 2009, and has emerged globally as a multidrug-resistant health care-associated fungal pathogen [1]. *Candida auris* usually colonizes on human mucosal and skin surfaces, as an opportunistic pathogen [2]. *Candida auris* often cause serious infections of the bloodstream, gut and other sites [3]. Reduced host immunity increases the risk for development of opportunistic infections due to *Candida auris* [4]. *Candida auris* could be resistant to several antifungal drugs. *Candida auris* could be making treatment ineffective and causing death rates can reach 60% [5]. *Candida auris* infection is now a constant threat to public health and socio-economic development because of its rapid spread in healthcare settings, with potential to cause outbreaks associated with higher mortality all over the world [6]. Learning about *Candida auris* infections-associated information including introduction, epidemiology, risk factor, symptoms, the susceptible person, treatment and prevention can help people to prevent *Candida auris* infections better. These detailed information will give people a better understanding of the risks and the actual impact of *Candida auris* infections.

In the twenty-first century, with the increasing popularity of information, people are increasingly tending to use the internet to obtain the health information [7]. And the Internet is becoming a significant and convenient source for information for patients and their families [8]. YouTube™ (http://www.youtube.com) as the largest free video-sharing website with over one billion users, a daily view count in the billions and more than 300 h of videos contents uploaded every minute [9]. YouTube™ is increasingly used to search health information [10]. Obviously, the importance and educational value of YouTube™ videos have been underestimated. Several studies have assessed health-related videos on YouTube™ such as oral leukoplakia, Diabetic Foot Care, Botulinum Toxin for Bruxism [11–13]. However, given the uncontrollable nature of information sources, there are significant risks associated with poor, incomplete and incorrect health information dissemination [14, 15]. Therefore, there is a need for a continuous critical assessment of the quality of YouTube™ health-related videos.

To our knowledge, no one has assessed the quality of *Candida auris* infection education videos on YouTube™. Thus, this study aims to evaluate the content, reliability and quality of YouTube™ videos regarding *Candida auris* infection, and to identify whether it is a useful resource for people.

## Methods

### Ethics statement

This study was exempt from Institutional Review Board approval of the study institution since it involved the use of public access data only.

### Search strategy

Our methodology is based on previous study [16]. The study population was composed of all YouTube™ videos containing information about *Candida auris* infection on September 21, 2019. The follow search key terms were used: “*Candida auris* infection” and “*Candida auris*”. And the YouTube™ search was sorted by the “Relevance” option of videos, which is probably the most common option for users. The first 100 videos (20 videos/page, first 5 page) of each search result were selected, because users usually screen within the first 5 pages of a search result. We ignored all the advertisements in the search results and in the beginning of video.

### Inclusion and exclusion criteria

The inclusion criteria were: (1) available on August 21, 2019; (2) related to *Candida auris* in content; (3) in English.

The exclusion criteria were: (1) non-English; (2) videos had no accompanying audio; (3) irrelevant videos; (4) duplicate videos; (5) advertisements.

In addition, Videos with multiple sub-parts were counted as one video.

### Variables

The follow information was extracted for each of the videos: the title; length of the video (in minutes); total numbers of views, comments, “dislike”, “like”; days since upload. If information was missing because of the video publisher restrictions, they were not considered in the corresponding analysis. For each video, we recorded the source of upload, categorized as government/news agencies, Universities/professional organizations/non-profit, physician/physician groups, Stand-alone health information websites, Medical advertisement/for profit companies, Individual and other. Regarding the characteristics of the individuals appearing in a video as well as the primary protagonist(s) in each video, we categorized it as patients, patient’s family or caregiver, physicians, nurses, reporter, social individual and others. In addition, Video style was categorized as follows: “educational video”, “entertainment”, “news report”, “politics”, “personal experience and blog”, “interview” and “others”. The “educational video” was uploaded with the purpose of providing kinds of information about *Candida auris* infection. We also determined percentage positivity (defined as the number of likes divided by the total number of likes or dislikes of that video); likebility (like per day); viewing rate (view per day) and viewer’s interactions (defined as the number of likes minus the number of dislikes divided by total number of views of that video).

### Scoring system

Similar to other studies, a point-based rating tool was constructed to evaluate video quality and specific content [17]. The overall quality of all selected videos was assessed using The Global Quality Scale (GQS) which is a five-point scale that was used to assess the educational value of each video (Table 1**)** [18]. Seven specific contents of the videos were systematically evaluated, including introduction, epidemiology, risk factors, symptoms, susceptible population, treatment and prevention of *Candida auris* infection. According to whether each content was specifically discussed, the content was given 0 point (Not mentioned), 1 point (Briefly introduced) and 2 points (Introduced in detail) as described previously [19]. A total score of 19 was available and a qualitative rating was given based on the reviewer’s score: “poor”(0–6), “good”(7–13), or “excellent”(14–19).

**Table 1:** Description of the Global Quality Score Five-Point Scale Used to Evaluate Web Sites Containing Information on *Candida auris infection*

Global Score	Global Score Description
1	Poor quality, poor flow of the site, most information missing, not at all useful for patients
2	Generally poor quality and poor flow, some information listed but many important topics missing, of very limited use to patients
3	Moderate quality, sub-optimal flow, some important information is adequately discussed but others poorly discussed, somewhat useful for patients
4	Good quality and generally good flow, most of the relevant information is listed,but some topics not covered, useful for patients
5	Excellent quality and excellent flow, very useful for patients

Each video was evaluated independently by two viewers to determine video eligibility for study inclusion (Jiangqing Huang and Shengcen Zhang). All viewers were blinded to each other’s result. Disagreements (differ by three or more points) between viewers regarding the content scores or GQS scores were resolved by an arbitrator (Qirong xiao) who given the final scores.

Jiangqing Huang and Shengcen Zhang are microbiology fellows with an interest in *Candida auris* infection. Qirong Xiao is a graduate student in clinical medicine with subspecialty training in hematology department who is knowledgeable in treatment of bacterial infections. They were trained before assessing the quality of videos. They received a document with URL of videos and scoring criterion from Bin Li respectively. And they didn’t discuss any detail during the assessment process.

In addition to the scores given by the arbitrator, the scores given by the two viewers were averaged for the final results and statistical analysis.

### Assessment of reliability

The DISCERN tool was used to assess the reliability of the YouTube™ videos [20]. This is a a five-point scale based on five questions, and each question is answered as yes or no. Each yes was given 1 point, for a total possible score of 5 points. As shown in Table 2.

**Table 2:** DISCERN Reliability Tool (1 point per question if answered yes)

1. Are the explanations given in the video clear and understandable?	
2. Are useful reference sources given? (publication cited, from valid studies)	
3. Is the information in the video balanced and neutral?	
4. Are additional sources of information given from which the viewer can benefit?	
5. Does the video evaluate areas that are controversial or uncertain?	

### Statistical analysis

Statistical analysis was performed using SAS 9.4 Statistical Software. The Cohen’s kappa coefficient (κ) and the interclass correlation coefficient (ICC) were calculated to assess the interobserver agreement. Fisher’s exact test (two tailed), Kruskal-Wallis test, Pearson test and Mann-Whitney test were performed for data comparison. Only *p* < 0.05 was considered statistically significant.

## Results

**Fig. 1:**
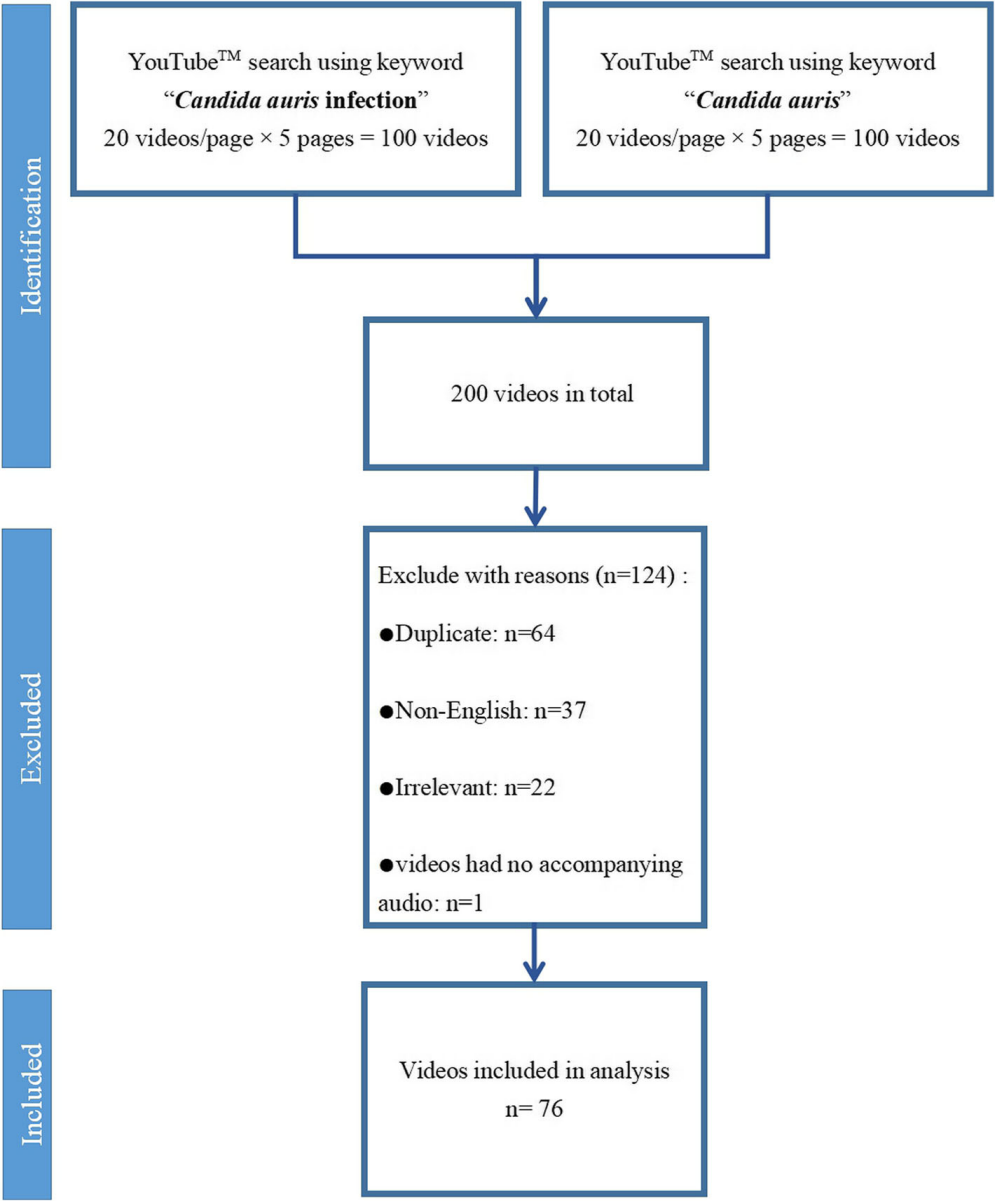
Details of videos included in the study

Using the search terms of “*Candida auris* infection” and “*Candida auris*”, 100 videos were screened for each of the two search terms. After screening using our inclusion and exclusion criteria, 124 videos were excluded, 76 (38.0%) videos were identified and selected for further analysis. Figure 1 presents the screening process.

Of the 76 videos evaluated, 40.8% (*n* = 31) were of poor, 40.8% (n = 31) were of good, and 18.4% (*n* = 16) were of excellent according to our Scoring system. The classification of relevant videos along with their attributes is described in Table 3.

**Table 3:** Comparison of the video parameters between the poor, good and excellent groups (median [quartile range])

	Poor (n = 31)	Good (n = 31)	Excellent (*n* = 14)	F	*p* value
length (min)	1.80 (1.10–4.40)	5.50 (2.50–14.50)	24.80 (13.20–54.00)	24.73	**< 0.0001**
like	6.00 (2.00–99.00)	19.00 (4.00–69.00)	22.50 (2.80–163.80)	0.19	0.8274
dislike	0 (0–3.00)	1.00 (0–5.00)	1.00 (0–24.50)	0.31	0.7323
view	838.00 (293.00–4826.00)	1130.00 (234.00–4567.00)	1997.00 (777.50–17,340.80)	0.33	0.7214
Posted days	166.00 (163.00–813.00)	164.00 (157.00–165.00)	166.50 (138.50–808.30)	3.35	**0.0407**
comments	2.00 (0–53.00)	9.00 (1.00–26.00)	4.0 (1.00–196.80)	1.12	0.3315
percentage positivity	0.98 (0.90–1.00)	0.96 (0.88–1.00)	0.96 (0.92–1.00)	0.79	0.4584
likebility	0.04 (0.004–0.61)	0.09 (0.02–0.42)	0.03 (0.02–1.00)	0.67	0.5125
view rate	2.25 (0.85–29.79)	6.33 (1.43–26.10)	2.97 (0.82–108.14)	0.91	0.4063
viewers’ interaction	0.01 (0.004–0.03)	0.02 (0.008–0.02)	0.01 (0.009–0.034)	0.27	0.7624
Total Views/day	47.12	31.96	13.94		
Total duration (seconds [%])	7902.00 (15.42)	17,971.00 (35.07)	25,372.00 (49.51)		
Total views (n [%])	372,376.00 (28.70)	571,214.00 (44.03)	353,710.00 (27.27)		

The mean DISCERN values among videos was 2.75 (SD = 1.59), and the score of DISCERN was positively correlated with the video score (*p* < 0.0001).

There were no statistically significant differences between groups in respect of the number of likes, dislikes, views, comments, percentage positivity, likebility, view rate, or viewers’ interaction. Length and post days were significantly associated with the classification (*p* < 0.0001 and *p* = 0.0407, respectively). The results of the correlation analysis showed that the length of videos was positively correlated with the video score (*p* < 0.0001). As shown in Table 4, Video category was associated with the quality of videos (*p* = 0.0023).

**Table 4:** Categorization of the videos according to Category, sources and characteristics [n (%)]

	Poor (n = 31)	Good (n = 31)	Excellent (n = 14)	Total	χ2	*p* value
Category						
Educational video	8	10	11	29	12.15	**0.0023**
Entertainment	0	0	0	0	–	–
News & Politics	7	6	0	13	3.37	0.1608
Interview & Blogs	15	15	3	33	3.38	0.1846
Others	1	0	0	1	1.47	0.4793
Source						
government/news agencies	10	13	4	27	1.00	0.6077
Universities/professional organizations/non-profit	9	6	4	19	0.89	0.6405
physician/physician groups	0	0	0		–	–
Stand-alone health information websites	3	2	3	8	2.34	0.3108
Medical advertisement/for profit companies	1	1	0	2	0.46	0.7930
Individual	8	9	3	20	0.29	0.8630
others	0	0	0	0	–	–
Characteristics						
patients	0	0	0	0	–	–
patient’s family or caregiver	0	0	0	0	–	–
physicians	11	16	9	36	3.59	0.1663
nurses	0	0	0	0	–	–
reporter	6	5	1	12	1.09	0.5809
social individual	6	8	2	16	0.86	0.6502
others	8	2	2	12	4.10	0.1110

In total, 29 (38.2%) of the videos were classified as “educational video”, 13 (17.1%) of were “news report”, 18 (23.7%) of were “personal experience and blog”, 15 (19.7%) of were “interview” and remaining one (1.3%) was “other” (Table 5). Table 5 and Table 6 were shown that the classification of videos according to category with details of other characteristics. A statistically significant difference was determined in favour of the video category in respect of the length of the videos, posted days and viewers’ interaction (*p* < 0.05). Significant differences were found in the source of videos and the characteristics of the individuals appearing in a video between the groups (*p* < 0.05).

**Table 5:** Detailed characteristics of videos based on category (median [quartile range])

	Educational video (*n* = 29)	News report (*n* = 13)	Personal experience and blog (*n* = 18)	Interview (*n* = 15)	Others (n = 1)	F	*p* value
length (min)	8.40 (2.70–24.80)	2.00 (0.70–2.30)	13.90 (4.50–20.60)	2.30 (1.70–4.00)	74.00	4.00	**0.0055**
like	22.00 (3.00–112.00)	19.00 (5.00–131.00)	14.00 (5.30–126.30)	6.0 (2.00–39.00)	38.00	0.36	0.8380
dislike	1.00 (0–4.50)	1.00 (0–13.00)	1.50 (0–3.50)	0 (0–6.00)	14.00	0.45	0.7705
view	1371.0 (265.0–4429.0)	1355.0 (777.5–19,700.5)	676.0 (170.5–2980.0)	1178.0 (214.0–3282.00)	6230.00	0.69	0.6045
Posted days	171.00 (156.50–715.00)	165.00 (150.00–607.50)	161.50 (148.80–165.00)	346.00 (164.00–816.00)	166.00	3.36	**0.0141**
comment	3.00 (0.50–19.50)	9.00 (1.50–134.00)	12.00 (1.00–69.00)	0 (0–26.00)	53.00	0.57	0.6871
percentage positivity	0.97 (0.94–1.00)	0.96 (0.88–1.00)	0.96 (0.87–0.99)	0.96 (0.91–1.00)	–	0.57	0.6837
likebility	0.03 (0.01–0.74)	0.12 (0.02–0.80)	0.092 (0.04–0.77)	0.007 (0.005–0.238)	–	0.29	0.8835
view rate	2.45 (1.10–20.02)	6.87 (2.30–119.65)	4.24 (1.15–18.06)	1.39 (0.49–20.02)	–	0.63	0.6429
viewers’ interaction	0.010 (0.008–0.022)	0.008 (0.003–0.019)	0.036 (0.019–0.061)	0.010 (0.004–0.013)	–	9.58	**< 0.0001**
Total Views/day	19.87	91.33	6.01	0.57	84.19		
Total duration (minutes [%])	29,014.00 (56.62)	1490.00 (2.91)	16,446.00 (32.09)	4221.00 (8.24)	74.00 (0.14)		
Total views (n [%])	576,377.00 (44.43)	136,081.00 (10.49)	84,074.00 (6.48)	494,538.00 (38.12)	6230.00 (0.48)

**Table 6:** Detailed characteristics of videos based on category

	Educational video (n = 29)	News report (n = 13)	Personal experience and blog (n = 18)	Interview (n = 15)	Others (*n* = 1)	Total	χ2	*p* value
Source								
government/news agencies	5	12	2	7	1	27	29.84	**< 0.0001**
Universities/professional organizations/non-profit	12	0	0	7	0	19	18.57	**0.0010**
physician/physician groups	0	0	0	0	0	0	–	–
Stand-alone health information websites	7	1	0	0	0	8	9.82	**0.0436**
Medical advertisement/for profit companies	2	0	0	0	0	2	3.33	0.5044
Individual	3	0	16	1	0	20	48.15	**< 0.0001**
others	0	0	0	0	0	0	–	–
Characteristics								
patients	0	0	0	0	0	0	–	–
patient’s family or caregiver	0	0	0	0	0	0	–	–
physicians	18	4	1	13	0	36	26.77	**< 0.0001**
nurses	0	0	0	0	0	0	–	–
reporter	2	7	2	1	0	12	17.31	**0.0017**
social individual	1	0	14	1	0	16	27.24	**< 0.0001**
others	8	2	1	0	1	12	12.60	**0.0134**

The Cohen’s kappa coefficient (κ) and the interclass correlation coefficient (ICC) were 0.6195 and 0.9498, respectively. The final score demonstrated good reliability.

## Discussion

With the widespread use of the Internet and social media, healthy people, patients with *Candida auris* and their family members often use online resource to get the health information. Inevitably, YouTube™ is used as a source of medical information as one of the largest video resource platforms in the world. Therefore, a large number of studies have assessed the quality of information on YouTube™, such as Schizophrenia, anterior cervical discectomy and fusion, breast self-examination, palliative care education and shoulder tests, but have shown considerable heterogeneity [21–25]. There are mixed reviews of YouTube™ as a source of information. To the best of our knowledge, this study is the first to evaluate the accuracy and the reliability of the content of YouTube™ videos about *Candida auris* infection. Our research aims to better understand the nature of people’s independent access to information regarding *Candida auris* infection on a large online media sharing platform.

The mean DISCERN values among videos included in this study was 2.75/5.00. The low score rates reflect a lack of structured, accurate, and reliable information regarding *Candida auris* infection on YouTube. And the score of DISCERN was positively correlated with the video score (*p* < 0.0001) indicated the reliability of the videos was positively correlated with the quality of videos.

In our study, the evaluated videos had over 1.2 millions views, total 25,153 likes, 1113 dislikes and total cumulative duration of 14.4 h and the videos garnered 7666 comments. These statistics demonstrated that most people use YouTube™ as a channel to learn about *Candida auris* infection and share their experiences and opinions.

Generally, the specific content of the included video is complex. Most of the videos listed risk factors or prevention. The results of this study had reference value for preventive healthcare [26]. Surprisingly, there are few discussion about treatment of *Candida auris* infection, which probably due to the seriousness of *Candida auris* infection, and the current treatment policy is limited [6].

Our research found the category of the videos was related to the length, posted days, source of videos and characteristics of the individuals appearing in a video. As shown in Table 5 and Table 6. Although 47 (38.2%) of the videos was “educational video”, it was not statistically significant with other categories in terms of quantity distribution. This indicated that individuals from different fields paid more attention when *Candida auris* infection broken out, and expressed their opinions and suggestions through YouTube™ [4]. “Educational video” and “personal experience and blog” videos usually had longer length than others (*p* < 0.05), and this may be because these videos contain more contents or a longer discussion of a point. In addition, other categories videos were different from “interview” videos in posted days, this may be because the cases of *Candida auris* infection had recently been widely reported in the United States, which had attracted the attention of many people [27]. In the area of the source of videos, not unexpectedly, “news report” videos, “educational video” videos and “personal experience and blog” videos was related to government/news agencies, Universities/professional organizations/non-profit and Stand-alone health information websites and individual, respectively. In terms of the characteristics of the individuals appearing in a video, not surprisingly, “educational video” videos and “interview “videos were connected with physicians, “news report” videos and “personal experience and blog “videos was associated with reporter and social individual, respectively. Results from these studies suggest that educational strategies should take it into consideration that whether the diverse audience recognizes the professionalism of a given protagonist, which could increase the credibility of the videos [28].

In this study, we found that YouTube™ videos about *Candida auris* infection could be used as a reliable resource for patients. More than half of the YouTube™ videos (59.2%, “good” and “excellent”) had better quality after videos being categorized as poor, good and excellent. The percentage of useful videos is higher than previous studies on other topics [29, 30]. This may be related to the widespread epidemic caused by *Candida auris* infection recently which lead to more concern about it [31]. However, our study found that there were no statistically significant differences between groups in respect of the number of likes, dislikes, views, comments, percentage positivity, likebility, view rate and viewers’ interaction, this demonstrated that the viewer could not distinguish the usefulness of the information, which is consist with previous study [24]. In this study, we found that “excellent” videos had the longest length (*p* < 0.0001) and DISCERN scores were positively correlated with the video score (*p* < 0.0001). Indeed, many previous studies reported that the videos with a length over 10 min could dissuade some viewers [30, 32]. Therefore, proper video duration was critical to the spread of health information. Moreover, our results showed that “excellent” videos was significantly associated with the “educational video” videos, but the number of views for the top three most useful videos was relatively low. And videos spreading the correct and significant information about *Candida auris* infection was special important based on the fact that this infectious disease has caused huge losses [33]. Previous study reported that the “entertainment” videos may got more views [19]. Therefore, this result suggested that the entertainment enhancement of videos may attract viewers to browse those videos.

There are several limitations to our study. First, this was a cross-sectional study that highlighted only the availability of information on *Candida auris* infection at that time. YouTube™ search results were dynamic because many videos are uploaded or removed every day. Second, the videos included in this study were all English-language videos which may cause some high quality videos to be excluded. Furthermore, this study only focused on videos on the YouTube™ platform and ignored that people may used other websites to obtain relevant information. In addition, this study lacked a standardized tool to assess health information, as there are no validated tools yet.

## Conclusions

In conclusion, a wide variety of information about *Candida auris* infection is available on YouTube™ and more than half of the YouTube™ videos in terms of *Candida auris* infection have better quality. Therefore, YouTube™ has striking potential to be an effective user-friendly learning interface for people to obtain information of *Candida auris* infection.

## Data Availability

The datasets used and/or analysed during the current study are available from the corresponding author on reasonable request.

## Abbreviations

GQS: The global quality scale
URL: Uniform resource locator
κ: The Cohen’s kappa coefficient
ICC: The interclass correlation coefficient
SD: Standard deviation
